# A perspective on retina education through social media

**DOI:** 10.1186/s40942-020-00244-x

**Published:** 2020-09-22

**Authors:** Ricardo Luz Leitão Guerra

**Affiliations:** 1 Clínica de Olhos Leitão Guerra, Rua Catharina Paraguaçú, n08, Graça, Salvador, Bahia 40.150-200 Brazil; 2 Obras Sociais Irmã Dulce, Salvador, Brazil

**Keywords:** Social media, Education, Medical, Ophthalmology, Retina, Communication barriers

## Abstract

Since the beginning of the Internet, new ways of providing medical education have emerged. Social media networks are one of the most influential communication tools and allow content sharing, collaborative modification and interaction. Its relevance for teaching and learning in medical education has been extensively studied. These new media have also heightened the need for a new way of communication. The purpose of this article is to discuss the value of communication in medical education through social media and present an eight-year personal experience on this field.

## Background

The technological advancement over the last decade has caused an abrupt change in society [1, 2]. It has changed the way of buying, listening to music, ordering food, among others. This period, also known as the fourth industrial revolution, is evolving exponentially [2]. Speed is a major characteristic of this era and the possibilities of billions of people connected by high-performance smartphones are unlimited [2].

Since the beginning of the Internet, new ways of providing medical education have emerged. Research in this field has been evaluating these new methods for over two decades [3] and the applicability of e-learning in continuing medical education is well known [4].

Social media is one of the most influential communication tools and has reached worldwide [1]. It allows users to produce and share content without previous evaluations. The numbers are impressive: from a total of 7.7 billion people in the world, 3.8 billion use the Internet and 1.5 billion use short videos (stories) for daily communication [5].

Ophthalmology is omnipresent on social media networks [6]. For example, the American Academy of Ophthalmology twitter account has more than 27.7 thousand followers (data from April 2020—https://twitter.com/aao_ophth). Physicians' social network accounts are generally used as a form of online presence, to initiate discussions and disseminate scientific research findings [6].

The purpose of this article is to discuss the value of communication in medical education through social media and present a personal experience on this field.

## Overview of medical education through social media

Social media platforms such as collaborative modification websites (Wikis), social networking sites, podcasts, blogs and microblogs, allow educational content sharing in an interactive environment [7]. Twitter, Facebook (FB), podcasts and blogs are described as the most frequently used platforms for medical education [7, 8].

Regarding education methods, Twitter, FB and blogs are often used to complement case-based teaching and to discuss research results. Moreover, Wiki platforms and Podcasts provide easy access to educational content [7, 8].

The benefit of free and easily accessible educational content has its risks and the reliability of educational material shared through social media should be analyzed carefully to avoid misinformation [9].

Despite the description of social media relevance as a teaching and learning tool in medical education [1, 10], there is strong evidence questioning these results [7, 8, 10]. Details of how students use social media for education and the lack of reliability and validity of the instrument used for data collection are pointed as weaknesses of these studies [7, 8].

In the communication process, social media networks are the media in which the message will be transmitted from the sender to the receiver. Effectively using social media for education requires constructing a message for the specific medium [11], however the communication method used for medical education in the previous mentioned research was not considered as a potential variable in those studies [7, 8, 12].

## Improving communication: a game changer

The communication process consists of constructing a message to be: receptive, understood correctly, accepted, and one that provokes a reaction. However, to achieve this goal, the receiver’s comprehension ability must be considered [13].

Each social media platform requires a particular way of communication, for example, a blog uses conventional text while podcasts uses audio. The world is changing and contemporary culture is mainly image-oriented [13]. Nowadays, platforms that allow image sharing such as FB and Instagram (IG) are the most popular networks worldwide [14]. Furthermore, including a picture increases the impact of publications on Twitter by 35% [11].

Communication through images requires less labor, time and fewer skills to understand than reading a text and it also reduces the possibility of misunderstanding the message [13]. It uses human beings’ inherent ability to promptly identify, process and retain images [15]. On the other hand, building an image-based message requires more time and skills.

The need for an effective way for scientific communication to fit social media network requirements was observed in 2016 by Dr. Andrew Ibrahim, Creative Director at *Annals of Surgery*, who introduced Visual Abstracts (VAs) [11, 15]. VAs are described as a “*single-page, multiplane, visual explanation of the study’s story*” and *“is designed to rapidly, visually, and memorably communicate the core concept of the study”* [11].

VAs were not designed to replace conventional texts and, similar to text abstracts, the aim of a VA is to provide useful information to help the reader decide if the full article is worth reading [11, 15]. The results of a case–control crossover study showed that VAs are associated with higher levels of research dissemination (7.7-fold increase in impressions and 2.7 increase in accessing the full article link) [16].

There are various ophthalmology educational social media accounts, however the vast majority of these accounts construct the message using long texts to describe an image, which is exactly the same as conventional textbooks. Table 1 presents a list of ten outstanding IG educational accounts and their communication particularities. Understanding the benefits of image-based communication might improve these accounts to an unlimited range.

**Table 1:** Outstanding Instagram educational accounts and its communication particularities

Instagram profile	Owner / Editor	Main communication method	Strengths
Retina.review	Not specified	Clinical photography + text description	Wide range of cases
The_eye_doctor	Not specified	Clinical photography or video + text description	Original content. Texts tends to be short and divided into topics
Retina.rocks	Not specified	Clinical photography + text description	Wide range of cases
Surgeonretina	Rajesh Rao	Clinical photography + Quiz	Pictures are often from recently published articles
Nyeye	Nikola Ragusa	Clinical photography + Question	Wide range of cases and interaction
Retina.vitreo	Caio Franco	Clinical photography + text description	Hi-quality images and multimodal evaluation
Jordical_retina	Jorge Calzada	Clinical photography or video + text description	Original content. Literature data versus personal experience is shared in a responsible way
Instambulretina	Not specified	Clinical photography + text description	Original content
Retinatips	Filipe Lucatto and Juliana Prazeres	Short videos explaining retinal surgery techniques	Innovative method to share surgical content
Retinaldystrophies	Not specified	Clinical photography + text description	Original content

## Personal experience with social media education

@retinography (https://www.instagram.com/retinography) is a social media profile that shares retina educational content and has a particular way of presenting it. To date, it is a non-profit free access account, which started in 2012 on IG and extended to other social media networks, such as FB, Twitter and YouTube.

Despite individuals having the first impression that the differential of the page is high quality images and videos, it is in fact the form of communication (how the message is constructed) that uses a “mixed, predominantly visual discourse” developed for adapting to the particularities of social networks that share images.

Short videos highlighting key-points of a disease, produced with this concept, are the most popular media shared on the @retinography social media networks. An additional movie file shows an example of this (see Additional files 1 and 2).

There are no language boundaries for images and this way of communication has enabled reliable scientific information to be shared with ophthalmologists all around the globe, transposing the alphabet barrier. For example, posts on FB reach thousands of people in more than 40 different countries. Moreover, Moscow is the second city in the world with more people following the @retinography IG account. Full IG and FB metrics are presented in an additional file (see Additional file 3).

Following this trend in communication has already enhanced my way of teaching and producing digital content beyond social media networks. Introducing this concept in a recently published iBook showed outstanding results. Forty-eight hours within publication, it was downloaded one thousand times in 41 countries. iBook metrics are fully presented in an additional file (see Additional file 3).

## Final considerations

Education through social media networks is evolving rapidly as new platforms and possibilities emerge. In spite of the limitations concerning education through social networks, this method should not be disregarded and the form of communication can be an important source of bias in the studies.

Ophthalmology is a mostly-visual medical specialty and image-based communication seems more logical. Despite the strengths of the method presented, as in VAs, it is a complementary method of teaching and does not replace the traditional methods used by long texts.

## Supplementary information

**Additional file 1.** Short video highlighting key-points of a Vitelliform Macular Distrophy.

**Additional file 2.** Short video highlighting key-points of a Central Retinal Artery Occlusion.

**Additional file 3.** Full Instagram, Facebook and iBook metrics.
