# Supplementary material for: A perspective on retina education through social media
Source: Int J Retina Vitreous. 2020 Sep 22;6:44. doi: 10.1186/s40942-020-00244-x (PMC7510150; doi:10.1186/s40942-020-00244-x)
Supplement: Supplementary file 3 — Additional file 3. Full Instagram, Facebook and iBook metrics. [file 40942_2020_244_MOESM3_ESM.pdf]

INSTAGRAM:

Data from August 13, 2020

34K Followers

COUNTRY

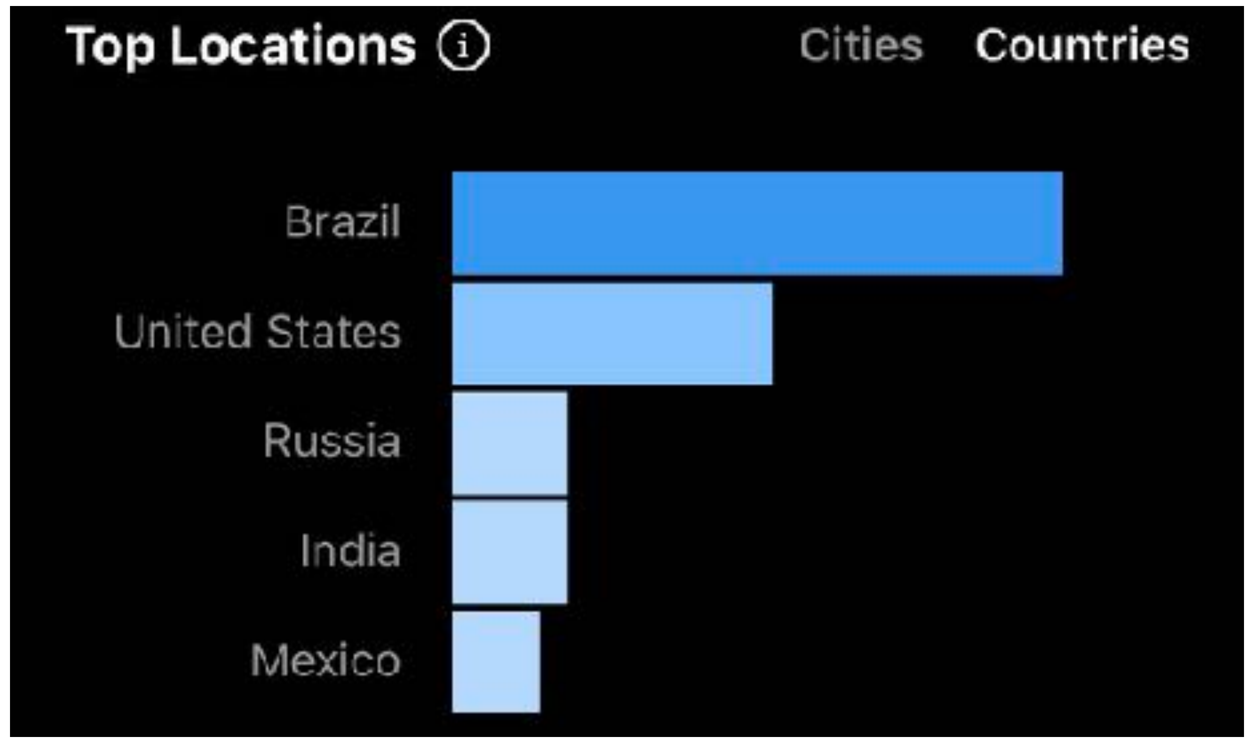

CITIES

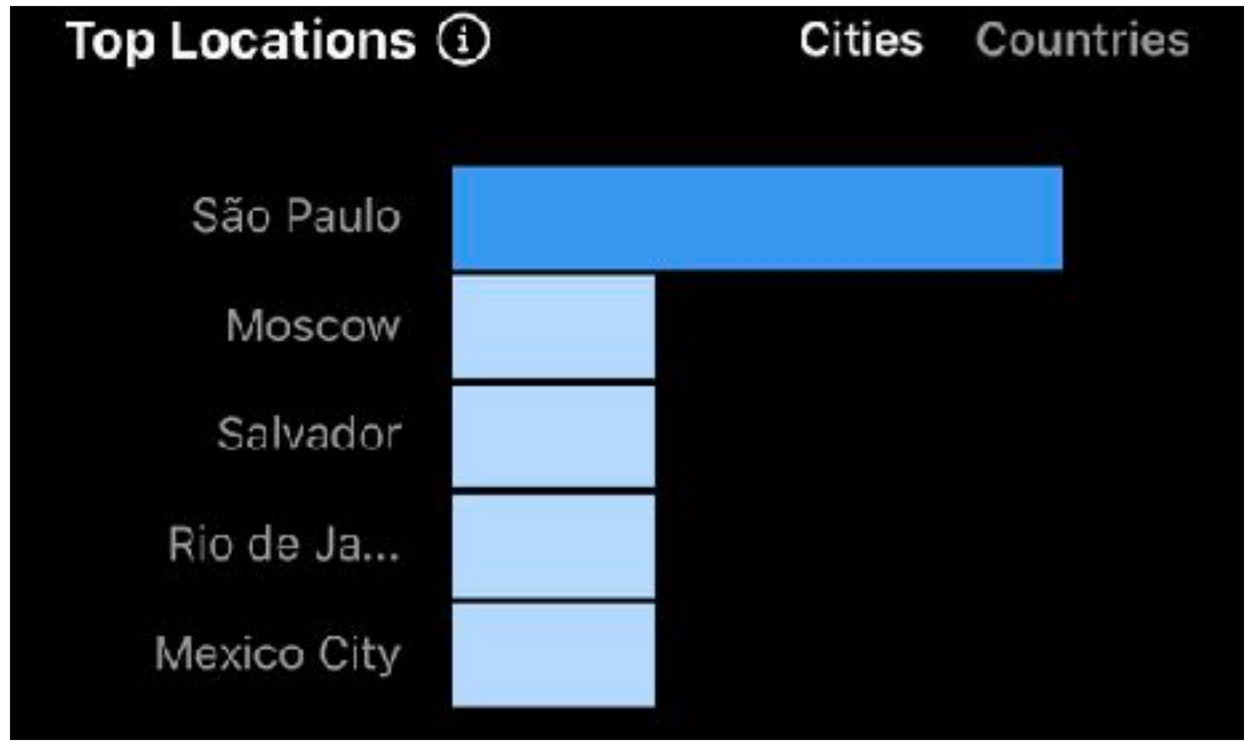

AGE

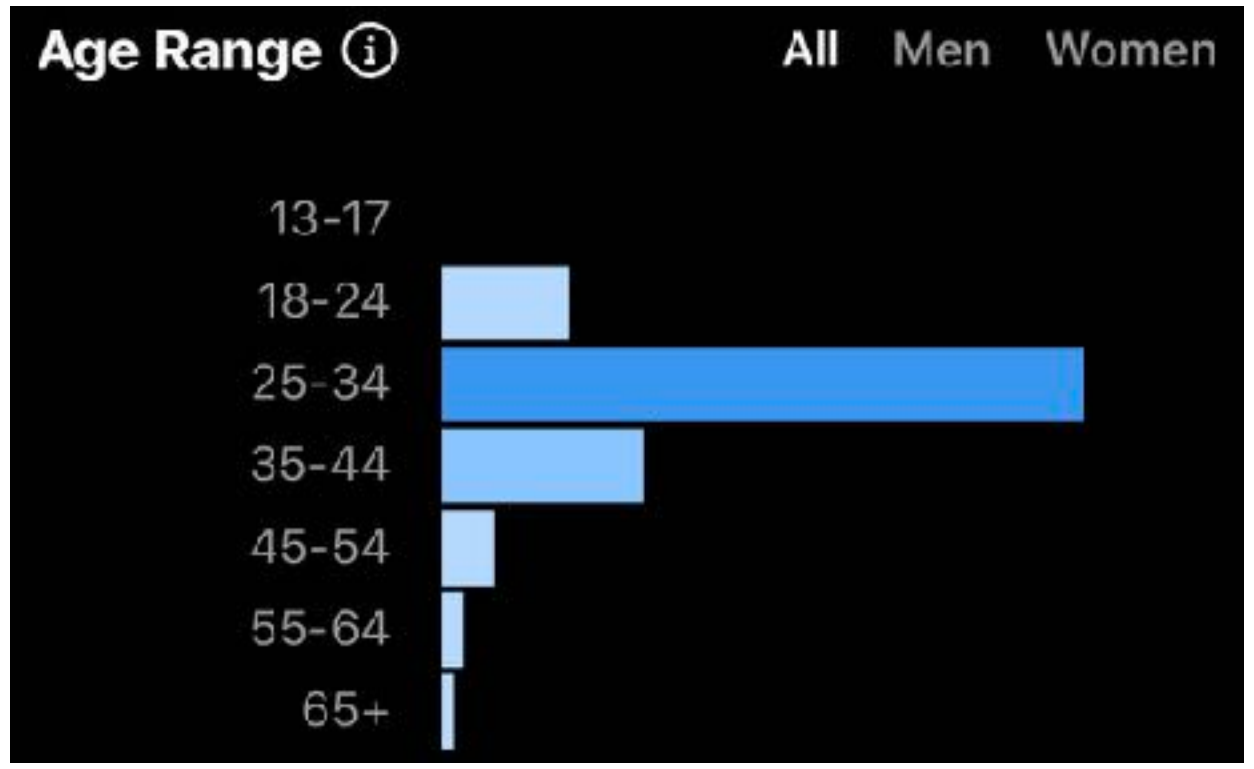

REACH

*Number of people who saw the content

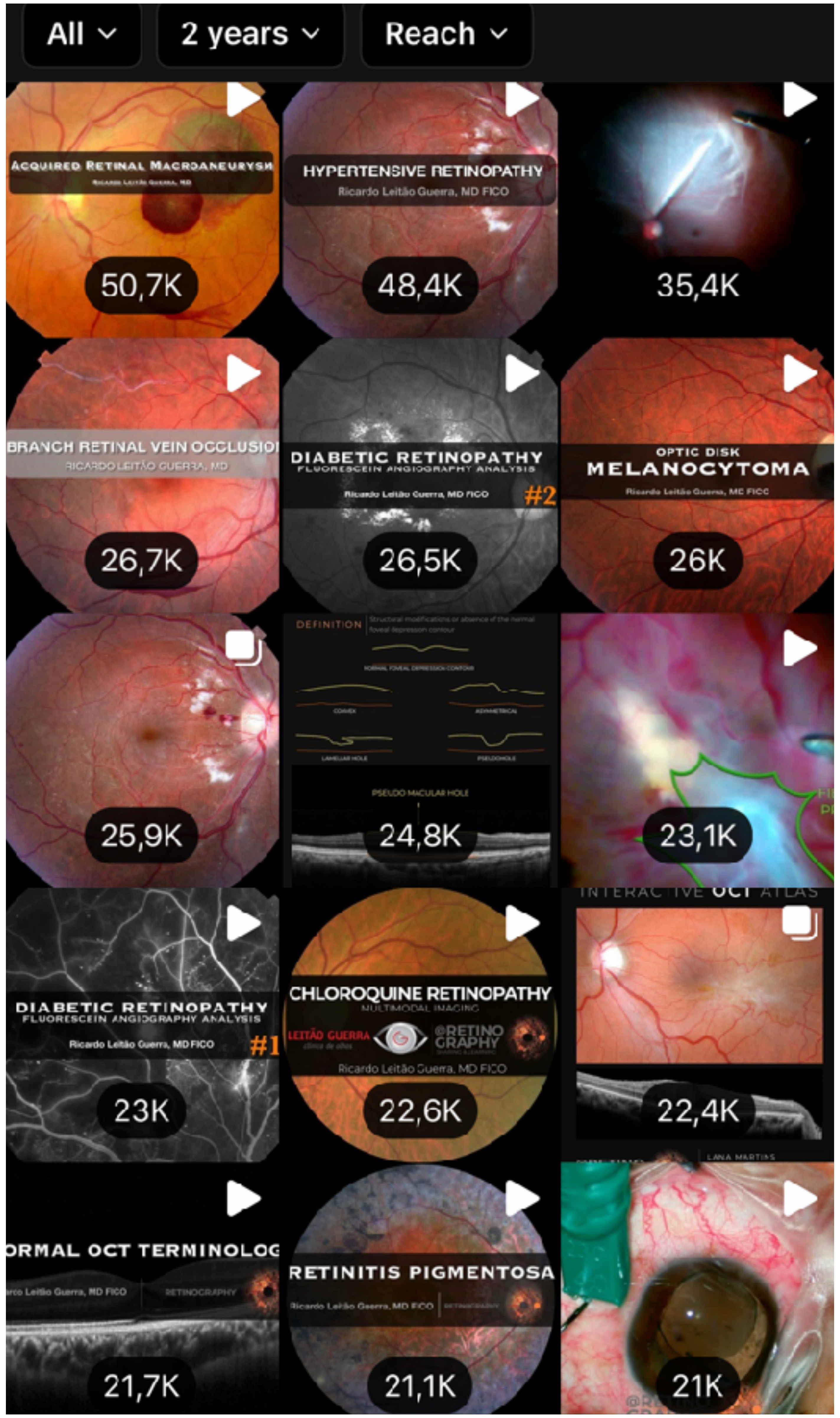

IMPRESSIONS

*Number of times the content was watched

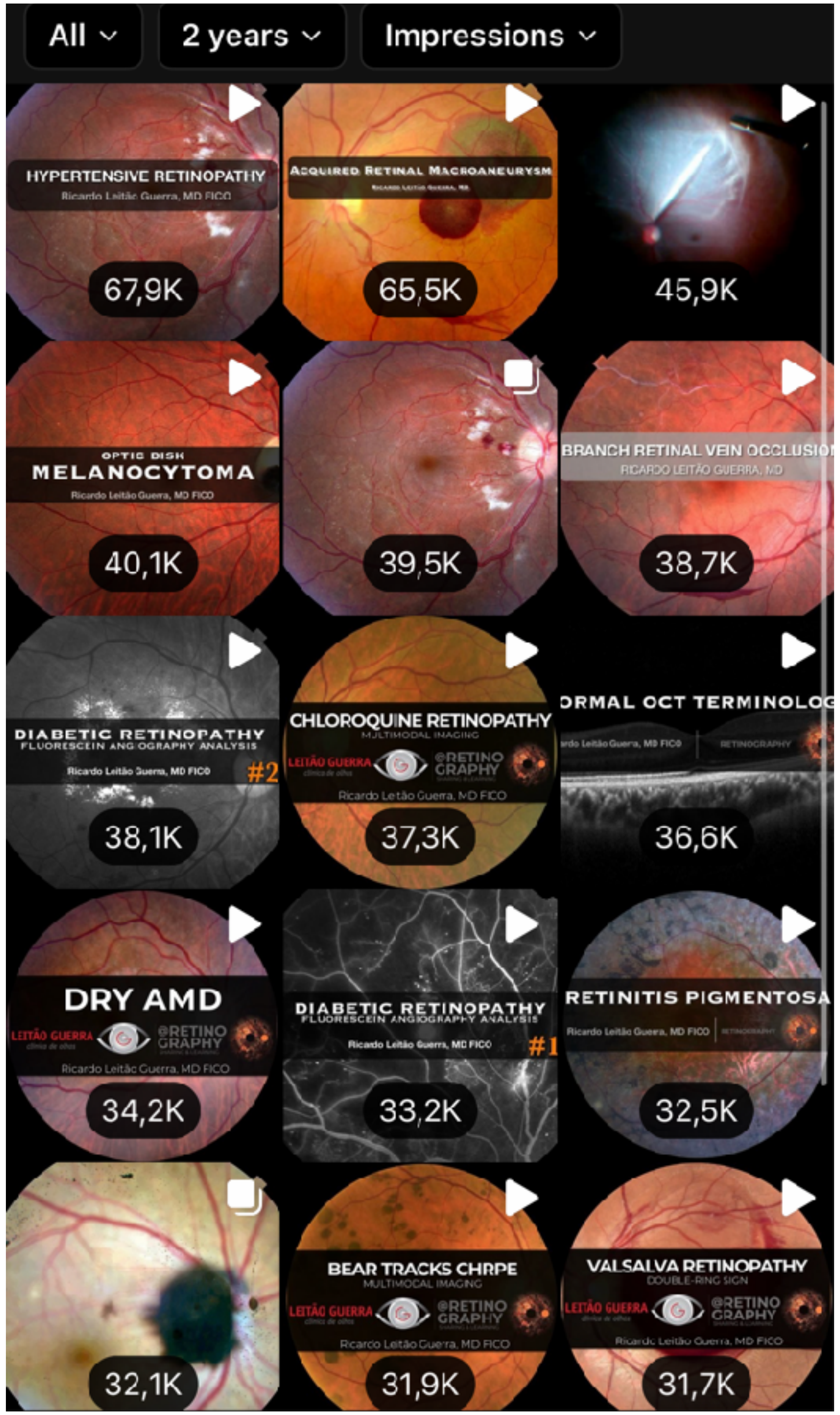

FACEBOOK:

Data from April 18, 2020

7,580 followers

The number of people who had any content from your Page or about your Page enter their screen screen, grouped by age and gender. This number is an estimate.

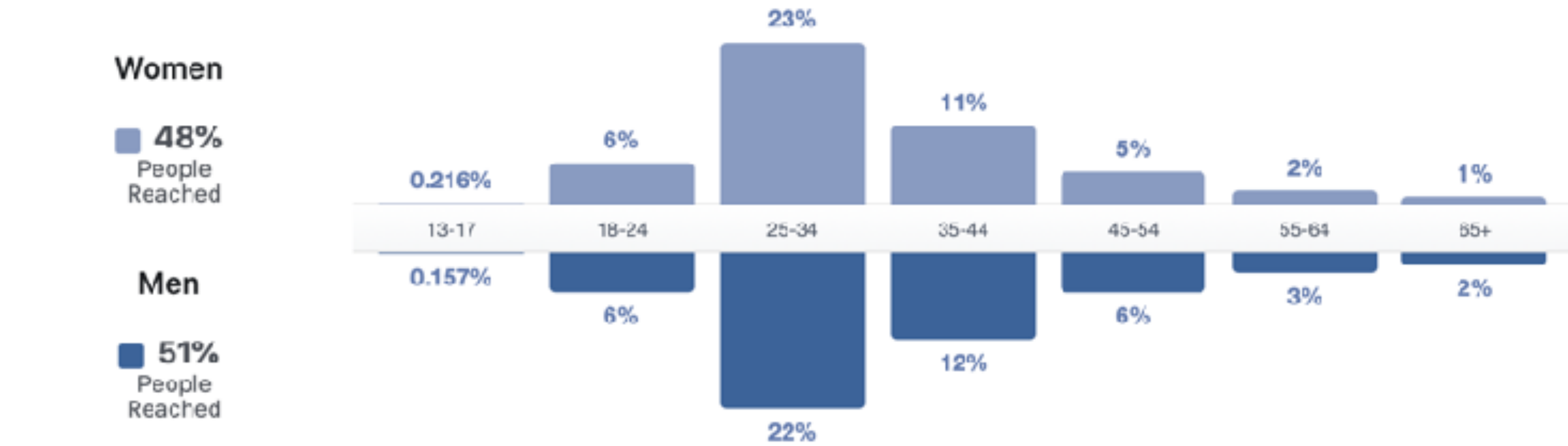

Country	People Reach...
India	14,414
United States of America	9,372
Brazil	4,063
Pakistan	3,990
Egypt	3,019
Ukraine	1,769
Italy	1,664
Romania	1,420
Bangladesh	1,416
Germany	1,409
Nepal	1,278
United Kingdom	1,164
Greece	1,086
Russia	1,072
Canada	963
Syria	947
Mexico	941

City	People Reach...
Delhi, India	1,151
Cairo, Egypt	1,126
Dhaka, Bangladesh	929
Lahore, Pakistan	920
Mumbai, India	725
Chennai, India	708
Athens, Greece	706
Karachi, Pakistan	657
Kathmandu, Nepal	616
Bucharest, Romania	616
Bangalore, India	589
São Paulo, SP, Brazil	577
Munich, Germany	564
Hyderabad, India	533
Lusaka, Zambia	530
Kolkata, India	504
Moscow, Russia	497

Language	People Reach...
English (US)	33,761
English (UK)	11,926
Portuguese (Brazil)	3,965
Arabic	3,768
Russian	3,214
Spanish	2,380
French (France)	2,062
Italian	1,673
Romanian	1,157
German	1,125
Greek	976
Vietnamese	837
Spanish (Spain)	706
Portuguese (Portugal)	605
Hindi	410
Polish	400
Ukrainian	366

Reach: Organic / Paid   Post Clicks   Reactions, Comments & Shares

Published	Post	Type	Targeting	Reach	Engagement	Promote
03/25/2020 2:33 PM	Free iBook. Download at: https://books.apple.com/br/book/epi			35.5K	3.5K 565	Boost Post
03/21/2020 7:59 PM	The COVID-19 pandemia has reduced the fellows clinical and			37.4K	4.9K 788	Boost Post
01/09/2020 1:02 PM	Four-point Scleral Fixation using 5-0 Prolene + akreos			26.7K	3.1K 690	Boost Post
10/28/2019 2:05 [illegible] Public	@guerraneto47 #retina #oftalmo #ophthalmology #oftalmologia			37K	2.3K 719	Boost Post
10/23/2019 8:52 PM	#valsalva #retina #oftalmo #ophthalmology #oftalmologia			37.4K	3.3K 941	Boost Post
10/08/2019 9:21 PM	PVR peeling under silicone oil 😏 #retina #oftalmo #ophthalmology			23.8K	1.6K 436	Boost Post
10/04/2019 9:34 AM	🐻 👣 #chrpe #retina #oftalmo #ophthalmology #oftalmologia			36.7K	4K 937	Boost Post
09/13/2019 5:36 AM	⏰ #amd #retina #oftalmo #ophthalmology #oftalmologia			23.8K	2.3K 700	Boost Post
08/26/2019 6:00 AM	#retina #oftalmo #ophthalmology #oftalmologia #oftalmologia			26.6K	2.9K 802	Boost Post
08/24/2019 4:15 AM	#retina #oftalmo #ophthalmology #oftalmologia #oftalmologia			58.1K	7K 1.9K	Boost Post
07/18/2019 9:44 PM	Fluorescein angiography findings in diabetic retinopathy #1			36.3K	2.7K 725	Boost Post
07/17/2019 11:09 PM	Hypertensive retinopathy! #retina #ophthalmology #oftalmo			78.5K	9.3K 1.9K	Boost Post

iBook

Data from April 18, 2020

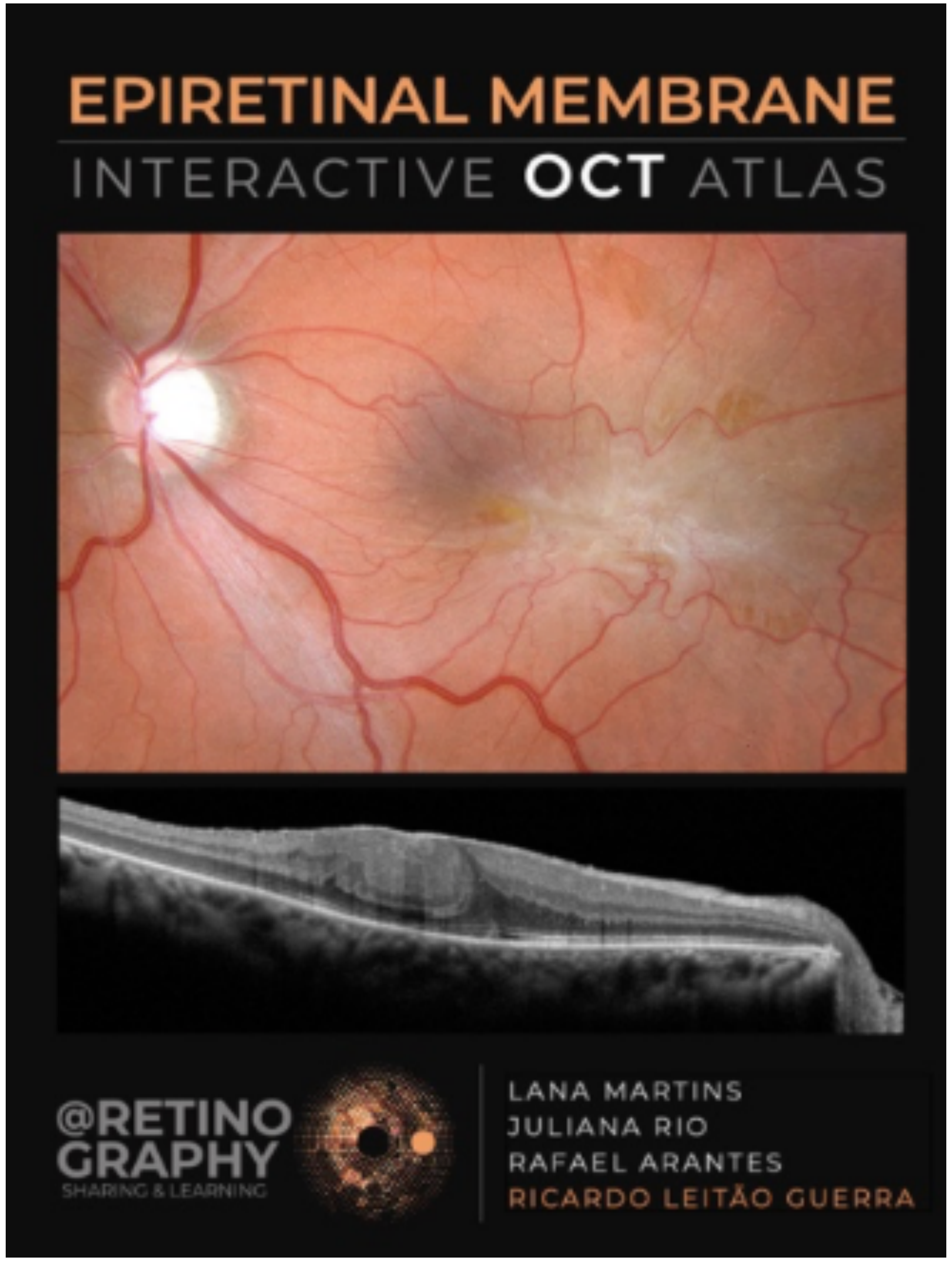

2.01K Units

Mar 23 - Apr 18, 2020

	Territory	Units
1	Latin America and The Caribbean	1.28K
2	Europe	413
3	USA and Canada	293
4	Asia Pacific	25

	Territory	Units
1	Brazil	1.11K
2	Mexico	38
3	Argentina	23
4	Chile	22
5	Colombia	17

	Territory	Units
1	Italy	103
2	United Kingdom	49
3	Germany	40
4	France	37
5	Romania	33

Territory	Units
Australia	21
Japan	2
New Zealand	2

	Territory	Units
1	United States	267
2	Canada	26
